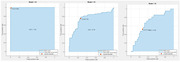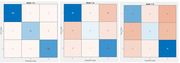# Volumetric imaging analysis using deep learning classifier for various stages of dementia

**DOI:** 10.1002/alz.092019

**Published:** 2025-01-09

**Authors:** Eva YW Cheung, Henry Mak, Yat Fung Shea, Patrick Ka‐Chun Chiu

**Affiliations:** ^1^ The University of Hong Kong, Hong Kong Hong Kong; ^2^ Tung Wah College, Hong Kong Hong Kong; ^3^ Queen Mary Hospital, Hong Kong Hong Kong

## Abstract

**Background:**

This study aimed to apply deep learning for various stages of dementia classification.

**Methods:**

The ADNI database and OASIS database were used, where ADNI 21 centers with total 406 images (69AD, 202MCI and 135HC) were used as training and validation, and ADNI 4 centers with 176 images (28AD, 91MCI and 57HC) were used as testing, and another 176 images (28AD, 91MCI and 57HC) from local memory clinic were used as local testing. The 39 brain regional volumes were segmented and calculated using Freesurfer. Ten‐fold cross validation were performed to minimize the chance of overfitting. Four classifiers, including decision tree (DT), ensemble classifier (EC), support vector machine (SVM), K‐nearest neighbors algorithm (KNN) were used to build the model in the MATLAB classifier toolbox.

**Results:**

The receiver operating characteristics (ROC) curves showed that the SVM achieved the best and balanced result, with AUC =1.00, 0.76 and 0.68; with 98.0%, 74.3% and 59.7% accuracy in training, testing and local testing respectively. This study showed that the training set attained the highest accuracy, while it dropped in testing data and local data. It highlighted the importance of local data testing prior to the application.

**Conclusion:**

The SVM can differentiate AD, MCI from HC with satisfactory result. The application is practical and can be fully automatic, which can be used for dementia screening. Further studies should be conducted to fine‐tune the models for improving the accuracy in local dataset, prior to the application in local application.